# Preliminary Observations of Spatial Imbalance between Lymphangiogenesis and Angiogenesis within Carotid Atherosclerotic Plaques: A Pilot Histopathological Study

**DOI:** 10.3400/avd.oa.25-00157

**Published:** 2026-04-01

**Authors:** Yohei Tateishi, Hiroaki Otsuka, Daiji Torimura, Aya Yamashita, Yuki Tomita, Takuro Hirayama, Tomoaki Shima, Shunsuke Yoshimura, Teiichiro Miyazaki, Reiko Ideguchi, Yuki Matsunaga, Hajime Maeda, Junya Fukuoka, Yoichi Morofuji, Tsuyoshi Izumo, Akira Tsujino

**Affiliations:** 1 Department of Neurology and Strokology, Nagasaki University Hospital, Nagasaki, Nagasaki, Japan; 2 Department of Clinical Neuroscience, Unit of Clinical Medicine, Nagasaki University Graduate School of Biomedical Sciences, Nagasaki, Nagasaki, Japan; 3 Department of Radioisotope Medicine, Atomic Bomb Disease Institute, Nagasaki University, Nagasaki, Nagasaki, Japan; 4 Department of Neurosurgery, Nagasaki University Graduate School of Biomedical Sciences, Nagasaki, Nagasaki, Japan; 5 Department of Pathology Informatics, Nagasaki University Graduate School of Biomedical Sciences, Nagasaki, Nagasaki, Japan; 6 Department of Neurosurgery, Showa Medical University, Tokyo, Japan; 7 Department of Neurosurgery, Gifu University Graduate School of Medicine, Gifu, Gifu, Japan

**Keywords:** angiogenesis, lymphangiogenesis, atherosclerosis, carotid plaque, immunohistochemistry

## Abstract

**Objectives:**

Atherosclerotic plaque progression involves dynamic interactions between angiogenesis and lymphangiogenesis. This pilot, hypothesis-generating study aimed to explore the spatial balance between blood microvessels and lymphatic vessels in human carotid plaques.

**Methods:**

Carotid plaques from 4 patients who underwent carotid endarterectomy were examined histologically. Blood microvessels (CD31+) and lymphatic vessels (Podoplanin+) were quantified in severely and mildly thickened lesions. The blood microvessel-to-lymphatic vessel (BMV/LV) ratio was compared between regions, along with macrophage polarization (CD80+: M1 and CD163+: M2).

**Results:**

Severely thickened lesions exhibited a 15.5-fold higher density of blood microvessels than mildly thickened lesions (median 31 vs. 2 vessels; p = 0.012), whereas lymphatic vessel counts were comparable (median 31 vs. 32 vessels; p = 0.978). Consequently, the BMV/LV ratio was 16-fold higher in severely thickened lesions (1.203 vs. 0.121, p = 0.060). CD80-positive areas were larger in severely thickened lesions (0.395% vs. 0.078% area, p = 0.006), whereas CD163-positive areas were comparable between regions (1.036% vs. 0.215%, p = 0.154).

**Conclusions:**

Mildly thickened carotid atherosclerotic plaques demonstrated relative lymphatic predominance, whereas severely thickened lesions showed angiogenic predominance accompanied by increased M1 macrophage infiltration. These exploratory findings suggest that regional imbalance between lymphatic vessels and blood microvessels within individual plaques may contribute to localized plaque vulnerability.

## Introduction

Atherosclerosis is characterized by lipid accumulation and chronic inflammation within the arterial wall, leading to plaque formation and progression.^1,2)^ This process involves angiogenesis, which contributes to plaque growth and instability by facilitating the influx of inflammatory cells and lipids, and lymphangiogenesis, which promotes lipid clearance and modulates immune responses.^3,4)^ However, the spatial balance between angiogenesis and lymphangiogenesis across different regions of human carotid atherosclerotic plaques remains uncharacterized.

Recent studies emphasize on the importance of the microvascular environment within atherosclerotic plaques for the development of novel therapeutic strategies.^5,6)^ In particular, pharmacologic modulation of lipid metabolism and inflammation may influence plaque microvasculature, underscoring the need to understand these spatial patterns.^7,8)^ Therefore, this study aimed to investigate the spatial distribution of blood microvessels and lymphatic vessels within carotid artery plaques, hypothesizing that severely thickened lesions exhibit a spatial imbalance favoring angiogenesis, whereas mildly thickened lesions maintain lymphatic predominance, potentially contributing to regional plaque vulnerability.

## Materials and Methods

### Study population and specimen collection

A retrospective analysis was conducted on carotid artery plaque specimens obtained from 4 consecutive patients who underwent carotid endarterectomy following acute ischemic stroke at Nagasaki University Hospital in 2017. Demographic variables, vascular risk factors, relevant medical history, and current medications (antithrombotic agents and statins) were recorded. The carotid artery was selected because carotid endarterectomy provides relatively large human atherosclerotic specimens with pronounced intimal hyperplasia. The aim of this pilot study was not to investigate carotid-specific hemodynamic phenomena but rather to characterize the microvascular and inflammatory environment of intimal thickening, irrespective of its etiologic triggers.

Patients underwent routine imaging, including magnetic resonance imaging (MRI), MR angiography, duplex ultrasonography, and computed tomography angiography or digital subtraction angiography. High-resolution carotid MRI was performed in 3 patients (Cases 1, 3, and 4). Laboratory parameters (C-reactive protein, low-density lipoprotein and high-density lipoprotein, and estimated glomerular filtration rate) were measured preoperatively.

Endarterectomy specimens, comprising the entire intima and part of the media, were fixed in formalin, embedded in paraffin, sectioned at 5 μm, and stained with hematoxylin and eosin and Azan–Mallory. Only sections containing both severely and mildly stenotic lesions were included in the immunohistochemical analyses. In histological sections, a severely thickened lesion was defined as the region with the greatest plaque thickness, and a mildly thickened lesion was defined as a non-normal area with only slight intimal thickening and plaque formation in the same arterial segment.

The Institutional Review Board of Nagasaki University Hospital approved this study (no. 2024-10-13) on January 9, 2025, and waived the requirement for written informed consent owing to its retrospective design.

### Carotid plaque evaluation on MRI

Carotid plaque MRI evaluation was performed using the modified American Heart Association (AHA) classification.^9)^ A 3.0-T scanner acquired T1-weighted, T2-weighted, proton density-weighted, and time-of-flight images with a minimum voxel size of 0.25 × 0.25 × 1 mm^3^. Plaque components were identified on multi-contrast MRI according to published criteria.^10)^ A lipid-rich necrotic core was suspected when a plaque component appeared hypointense on T2-weighted and proton density-weighted images with isointense to slightly hyperintense signals on T1-weighted images relative to the adjacent muscle. Intraplaque hemorrhage was defined as a hyperintense area on T1-weighted and time-of-flight imaging.

### Histopathology and immunohistochemical analysis

Immunohistochemistry was performed using the following primary antibodies: CD31 (1:100; Agilent Technologies, Santa Clara, CA, USA) for blood endothelial cells; podoplanin (clone D2-40, ready-to-use; Roche, Basel, Switzerland) for lymphatic endothelial cells; CD68 (1:400; Agilent) for macrophages; CD80 (1:100; Abcam, Cambridge, UK) for M1-type macrophages; CD163 (1:200; Leica Biosystems, Wetzlar, Germany) for M2-type macrophages; and α-smooth muscle actin (ready-to-use; Leica Biosystems) for myofibroblasts. Digital images were analyzed using ImageJ software (version 1.53a; National Institutes of Health, Bethesda, MD, USA), and CD80- and CD163-positive areas were expressed as the percentage of plaque area.

### Regions of interest (ROI) and quantitative analysis

**Fig. 1:**
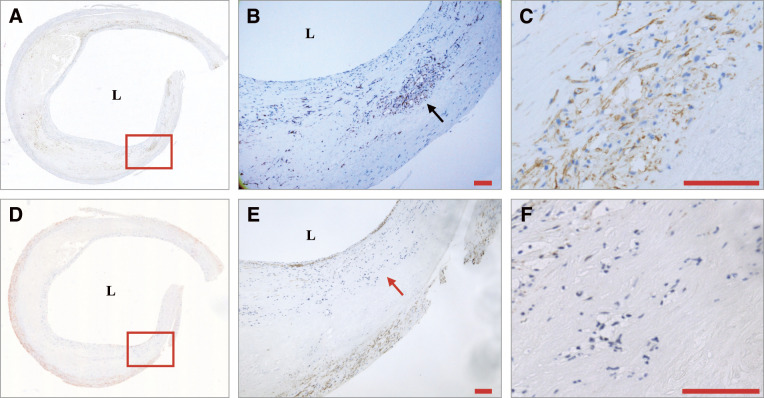
The transverse section of the internal carotid artery showing a mildly thickened lesion was stained with podoplanin (**A**). Magnified views of the rectangular region of interest in the plaque and the region indicated by the black arrow demonstrate podoplanin-positive luminal structures (**B**: ×40; **C**: ×800). The same section was stained with α-smooth muscle actin (**D**). Magnified view of the rectangular region of interest reveals an unstained area (red arrow) corresponding to a podoplanin-positive region (**E**: ×40; **F**: ×800).

For each specimen, 2 lesions were identified as severely and mildly thickened lesions. Within each lesion, an ROI corresponding to the entire plaque cross-sectional area, including the shoulder regions, was traced on digital images. All CD31- and podoplanin-positive luminal structures within this ROI were counted as absolute vessel numbers per lesion at high-power magnification (×800). Blood microvessels were defined as CD31-positive luminal structures typically containing red blood cells, and lymphatic vessels as podoplanin-positive luminal structures after excluding α-smooth muscle actin-positive structures to avoid misclassification of myofibroblast-rich structures (**Fig. 1**).

**Fig. 2:**
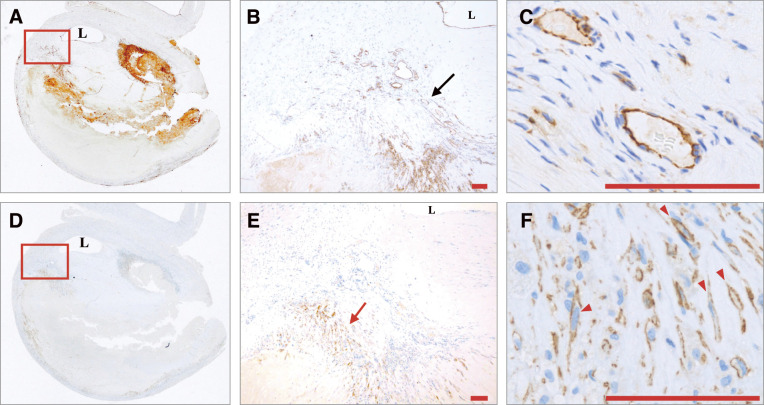
The transverse section of the internal carotid artery showing a severely thickened lesion was stained with CD31 (**A**). Magnified views of the rectangular region of interest in the plaque and the region indicated by the black arrow show CD31-positive luminal structures containing red blood cells (**B**: ×40; **C**: ×800). The same section was stained with podoplanin (**D**). A magnified view of the rectangular region of interest shows a podoplanin-positive area (red arrow) (**E**: ×40). Podoplanin-positive luminal structures occasionally contain mononuclear cells (red arrowheads) (**F**: ×800).

Plaque shoulders were defined as the transition zones between the fibrous cap and the adjacent relatively normal intima, which are considered mechanically and biologically vulnerable regions of the plaque.^11,12)^ For each shoulder, a predefined rectangular ROI (**Figs. 1** and **2**) was placed along this transition zone on the digital image, and CD31- and podoplanin-positive luminal structures within this ROI were identified and counted under high-power magnification (×800).

Thus, vessel counts in whole plaques represent the absolute number per cross-section, whereas vessel counts in plaque shoulders represent the number per predefined shoulder ROI. The blood microvessel-to-lymphatic vessel (BMV/LV) ratio was calculated for each lesion in the study.

CD80- and CD163-positive areas were manually delineated on digital images based on visual color discrimination without using a predefined automated threshold and expressed as the percentage of the plaque cross-sectional and shoulder areas. All measurements were performed by a single observer (T.M.) who was blinded to the clinical data and lesion classification. Inter-observer variability was not evaluated.

To ensure the validity and reliability of the findings, the vessels had to exhibit a clearly discernible lumen; tangential CD31- or podoplanin-positive profiles without a definite lumen or with ambiguous staining were not counted as vessels to minimize misclassification.

### Statistical analyses

Numerical variables were compared using the Wilcoxon signed-rank test, and categorical variables were analyzed using Fisher’s exact test. Data are presented as median (interquartile range [IQR]) or frequency (%). Matched-pair analyses were applied to the BMV/LV ratio, vessel counts, and CD80/CD163-stained areas between severely and mildly thickened lesions. All tests were 2-tailed, and p-values <0.05 were considered statistically significant. Analyses were performed using JMP Pro 18 (SAS Institute, Cary, NC, USA).

## Results

**Fig. 3:**
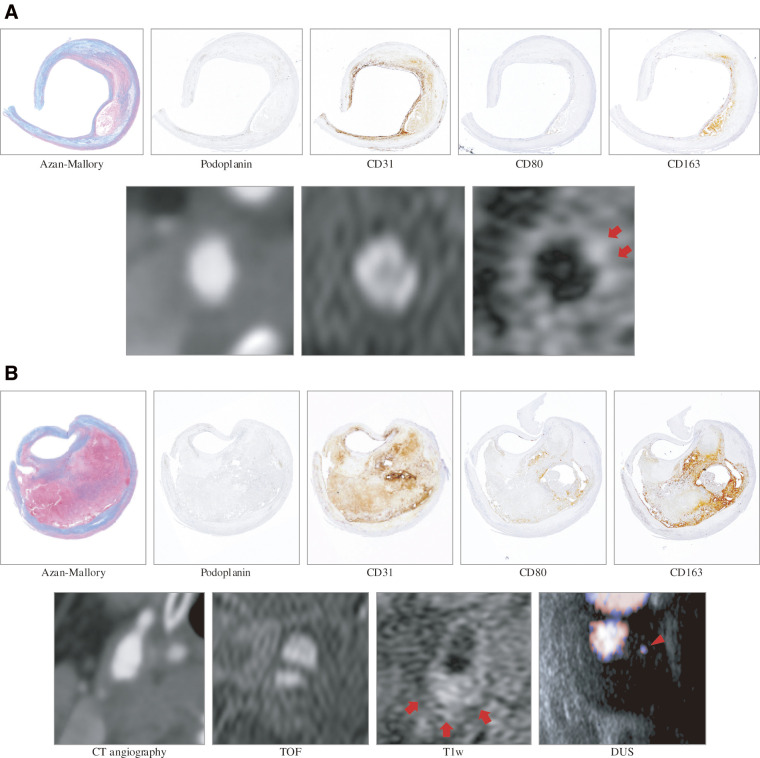
Histopathological and imaging findings of mildly and severely thickened lesions in Case 1. (**A**) In the mildly thickened lesion, T1-weighted magnetic resonance imaging shows a hyperintense area corresponding to lipid-rich necrotic core and intraplaque hemorrhage, as confirmed by histopathologic examination (red arrows). (**B**) In the severely thickened lesion, T1-weighted magnetic resonance imaging shows a hyperintense lesion throughout, corresponding to areas of lipid-rich necrotic core and intraplaque hemorrhage (red arrows). Duplex ultrasonography reveals the presence of blood flow signals within the plaque, which appear to correspond to a cluster of blood microvessels (red arrowhead). CT: computed tomography; TOF: time-of-flight; T1w: T1-weighted; DUS: duplex ultrasonography

Patient characteristics are presented in **Table 1**. Case 2 did not undergo an MRI. High-resolution carotid T1-weighted MRI revealed a high-intensity lesion in the severely thickened region in Cases 1, 3, and 4, consistent with lipid-rich necrotic core or intraplaque hemorrhage on histopathology, although not confined to CD31-, CD80-, or CD163-positive regions (**Fig. 3** and **Supplementary Figs. 1** and **2**).

**Table 1:** Clinical characteristics, laboratory findings, and imaging features of the 4 patients who underwent carotid endarterectomy

	Case 1	Case 2	Case 3	Case 4
Age, years	66	76	70	82
Sex	Male	Male	Male	Male
Vascular risk factor	HT, DM	HT, DM, HL, CKD	HT, HL	HT
Past medical history	IS	IS	IS, IHD	IS
Antithrombotic therapy prior to surgery	Aspirin	Clopidogrel	Aspirin	Aspirin
Statin use prior to surgery	Pitavastatin calcium	Pitavastatin calcium	Rosuvastatin calcium	No
Laboratory findings				
C-reactive protein (mg/dL)	0.14	0.03	0.03	1.02
Low-density lipoprotein (mg/dL)	112	59	62	56
High-density lipoprotein (mg/dL)	34	46	37	36
eGFR (mL/min/1.73 m^2^)	52.11	39.35	61.33	53.6
NASCET (%)	54	65	73	56
MRI findings of the carotid artery				
T1-weighted image	High	N/A	High	High
Time-of-flight image	High	N/A	Low	High
T2-weighted image	High	N/A	High	High
Proton density-weighted image	High	N/A	High	High
Modified AHA classification				
Mildly thickened lesion	Type IV–V	Type IV–V	Type IV–V	Type IV–V
Severely thickened lesion	Type IV–V	Type VI–V	Type IV–V	Type VI
Duplex ultrasonography of the carotid artery				
Blood flow signal within the lesion	P	N	N	N
Ulceration	P	N	N	N

MRI: magnetic resonance imaging; eGFR: estimated glomerular filtration rate; NASCET: North American Symptomatic Carotid Endarterectomy Trial; AHA: American Heart Association; HT: hypertension; DM: diabetes mellitus; HL: hyperlipidemia; CKD: chronic kidney disease; IS: ischemic stroke; IHD: ischemic heart disease; P: positive; N: negative

**Fig. 4:**
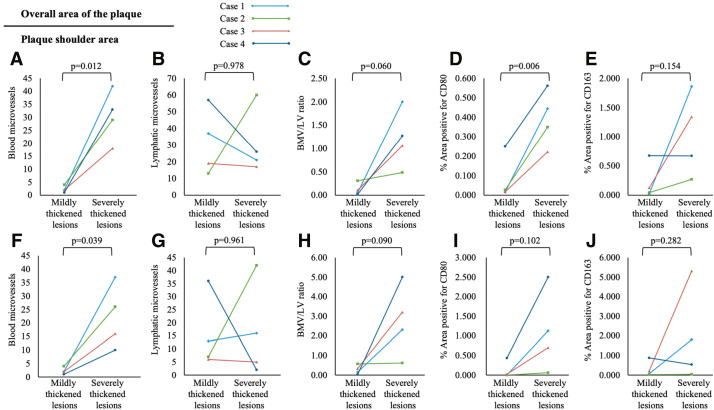
(**A**) Blood microvessel counts in the overall plaque area (severely vs. mildly thickened lesions: 31 vs. 2, p = 0.012). (**B**) Lymphatic vessel counts (31 vs. 32, p = 0.978). (**C**) BMV/LV ratio (1.203 vs. 0.121, p = 0.060). (**D**) CD80-positive area (0.395% vs. 0.078%, p = 0.006). (**E**) CD163-positive area (1.036% vs. 0.215%, p = 0.154). (**F**–**J**) Corresponding parameters in plaque shoulders (**F**: 22 vs. 2, p = 0.039; **G**: 16 vs. 16, p = 0.961; **H**: 2.783 vs. 0.272, p = 0.090; **I**: 1.098% vs. 0.114%, p = 0.102; **J**: 1.924% vs. 0.297%, p = 0.282). BMV/LV: blood microvessel-to-lymphatic vessel

Severely thickened lesions contained more blood microvessels than mildly thickened lesions, which showed only sparse microvessels (median, 31 vs. 2 vessels; p = 0.012). Lymphatic vessel counts were similar (31 vs. 32, p = 0.978), resulting in a higher BMV/LV ratio in severely thickened lesions than mildly thickened lesions (1.203 vs. 0.121, p = 0.060), with a similar trend in plaque shoulders (**Fig. 4**). In Case 2, abundant lymphatic vessels in the severely thickened lesion led to a relatively low BMV/LV ratio in both whole plaque and plaque shoulder, despite a higher ratio in the severely thickened lesion than in the mildly thickened lesion.

On low-power microscopy, CD80- and CD163-positive areas appeared side by side in both lesions (**Fig. 3** and **Supplementary Figs. 1** and **2**). The CD80-positive area was greater in severely thickened lesions than in mildly thickened lesions (0.395% vs. 0.078%, p = 0.006), whereas the CD163-positive area was comparable (1.036% vs. 0.215%, p = 0.154) (**Fig. 4**). Case-level CD80 and CD163 staining in severely thickened lesions is shown in **Supplementary Fig. 3**.

## Discussion

The results of this study demonstrated a spatial imbalance in the plaque microvasculature. Our preliminary observations may suggest a trend toward lymphatic vessel predominance in mildly thickened lesions, whereas angiogenesis is predominant in severely thickened lesions.

The spatial imbalance between angiogenesis and lymphangiogenesis, reflected by higher BMV/LV ratios in severely thickened lesions than in mildly thickened lesions, aligns with prior reports linking intraplaque angiogenesis to unstable phenotypes.^4,13)^ This intraplaque variation suggests that regions with preserved lymphatic dominance may help maintain lipid homeostasis, whereas regions with excessive neovascularization may exceed the remaining lymphatic drainage capacity and sustain inflammation, as supported by the accumulation of CD80+ macrophages in the region.^14–16)^ Therefore, the elevated BMV/LV ratio may serve as an indicator of local microvascular-inflammatory imbalance. Owing to the limited sample size and dynamic nature of microvascular remodeling, these findings should be regarded as exploratory and hypothesis-generating. Rather than indicating temporal stages of atherosclerosis, mildly and severely thickened lesions likely represent distinct local phenotypes within individual plaques that differ in microvascular balance and inflammatory activity. Our findings raise the possibility that pharmacologic modulation of lipid metabolism and inflammation can influence plaque lymphatic–angiogenic balance. Experimental data indicate that statins and proprotein convertase subtilisin/kexin type 9 loss-of-function improve lymphatic vessel growth or drainage capacity in cardiovascular models.^17,18)^ Thus, we speculate that the early initiation of lipid-lowering therapies, such as statins or proprotein convertase subtilisin/kexin type 9 inhibitors, may help preserve lymphatic predominance in mildly thickened lesions. Consequently, this approach potentially prevents progression toward angiogenic-dominant, severely thickened lesions. In addition to pharmacologic interventions, the modulation of lifestyle and environmental factors, including dyslipidemia, obesity, hypertension, smoking, and physical inactivity, may influence systemic inflammation and lipid metabolism.^19)^ Experimental data further suggest that obesity, metabolic dysfunction, and exercise can modulate lymphatic function and lymphangiogenesis in adipose tissue, skeletal muscle, and the heart.^20)^ However, direct evidence linking lifestyle interventions to intraplaque lymphangiogenesis in humans is currently lacking. These factors may also affect the plaque lymphatic–angiogenic balance, particularly in the earlier stages, where mildly thickened lesions show relative lymphatic predominance. Therefore, our observations hypothesize that comprehensive preventive strategies combining lifestyle modifications with aggressive risk factor control may help maintain or restore a more favorable lymphatic-dominant microvascular profile.

Notably, in Case 2, the patient who had chronic kidney disease showed unusually high lymphatic vessel numbers in the severely thickened lesion, a pattern that may reflect lymphatic expansion associated with kidney injury and chronic kidney disease.^21)^ This outlier case may have substantially influenced the calculated medians, underscoring the need to confirm these trends in large cohorts.

Our findings demonstrated the spatial coexistence of CD80+ (M1) and CD163+ (M2) macrophages in both lesion types. In severely thickened lesions, M1 dominance is consistent with their pro-inflammatory role, whereas persistent M2 macrophage presence may indicate incomplete resolution of inflammation.^22,23)^ The pronounced coexistence of M1 and M2 macrophages may further promote angiogenesis, with M1 macrophages mediating inflammation and hemoglobin-activated M2 macrophages releasing vascular endothelial growth factor to increase microvascular permeability and macrophage infiltration.^24)^ Case-level variation in M1/M2 ratios, including Case 4’s elevation in mildly thickened lesions, may underscore substantial intraplaque heterogeneity. Although animal models such as ApoE−/− mice suggest a temporal shift from M2 to M1 dominance, our human findings indicate that mixed M1/M2 patterns can coexist within single plaques, reflecting microenvironments of active inflammation and healed fibrous tissue that challenge simplified temporal interpretations.^25)^

T1-weighted imaging identified lipid-rich necrotic core or intraplaque hemorrhage consistent with histopathology, supporting MRI’s capacity to detect major plaque components.^26)^ However, MRI could not depict microvascular spatial imbalances, including CD31+/podoplanin + gradients, underscoring its limited microscopic resolution relative to histopathology. From a clinical and translational perspective, our pilot histopathological observations of regional lymphatic–angiogenic imbalance highlight the need for noninvasive or minimally invasive approaches that indirectly assess the microvascular and inflammatory milieu within atherosclerotic plaques. Contrast-enhanced ultrasound can visualize and quantify intraplaque neovascularization, with enhancement correlating with microvessel density and cerebrovascular events.^27)^ Positron emission tomography using ^18^F-fluorodeoxyglucose or tracers targeting integrin αvβ3 or fibroblast activation provides a noninvasive readout of plaque inflammatory activity and angiogenesis.^28,29)^ Moreover, 4D-flow MRI enables volumetric assessment of complex flow and wall shear stress linked to plaque burden and vulnerability.^30)^ However, no established noninvasive modality currently visualizes plaque lymphangiogenesis. Therefore, our histological findings may provide a conceptual framework for interpreting and refining future imaging biomarkers of plaque microvasculature and inflammation.

### Limitations

This study had some limitations. First, the evaluation of only 4 atherosclerotic plaques limits the ability to generalize spatial imbalance patterns. Second, the cross-sectional design prevents tracking the temporal evolution of these imbalances. Third, the absence of functional assays such as lymphatic drainage efficiency limits the mechanistic interpretation of spatial distributions. Fourth, in plaque shoulders, microvessels and lymphatic vessels were assessed within a single standardized high-power field per shoulder rather than across the entire shoulder area, which may not fully capture spatial heterogeneity. Additionally, we did not calculate area-normalized vessel densities. Fifth, all quantitative measurements were performed by a single experienced observer, and inter-observer variability was not assessed, which introduces a potential for observer-dependent bias despite blinded evaluation. Finally, CD31 staining did not distinguish between newly formed and pre-existing microvessels in the present study. Therefore, our assessment of angiogenesis reflects total microvascular density rather than definitive neovessel formation.

## Conclusion

In this small pilot study, we observed a spatial imbalance in the microvasculature within carotid plaques, with mildly thickened lesions showing lymphatic dominance and severely thickened lesions showing angiogenic dominance. This spatial polarization appears to correlate with macrophage activation gradients and features linked to plaque instability. These exploratory findings raise the possibility that therapeutic strategies targeting lymphatic–angiogenic crosstalk can help modulate plaque behavior, depending on the local microvascular environment. Future studies with larger cohorts are required to determine whether this spatial imbalance contributes to plaque vulnerability.

## Supplementary Materials

Supplementary Fig. 1 Histopathological and imaging findings of mildly and severely thickened lesions in Case 3. (**A**) The mildly thickened lesion contains a lipid-rich necrotic core, which appears slightly hyperintense on T1-weighted images. (**B**) In the severely thickened lesion, a lipid-rich necrotic core predominates, while intraplaque hemorrhage was minimal. The lesion appears slightly hyperintense on T1-weighted images and low intensity on time-of-flight images. CT: computed tomography; TOF: time-of-flight; T1w: T1-weighted; DUS: duplex ultrasonography

Supplementary Fig. 2 Histopathological and imaging findings of mildly and severely thickened lesions in Case 4. (**A**) The region containing both intraplaque hemorrhage and a lipid-rich necrotic core corresponds to hyperintense lesions on time-of-flight and T1-weighted images. (**B**) In the severely thickened lesion, part of the specimen appears disrupted near the region of calcification. The area where intraplaque hemorrhage and proliferated collagen fibers are intermixed demonstrates hyperintensity on time-of-flight (red arrows) and T1-weighted images (red arrowheads). CT: computed tomography; TOF: time-of-flight; T1w: T1-weighted; DUS: duplex ultrasonography

Supplementary Fig. 3 The graphs illustrate the percentage of CD80- and CD163-stained areas relative to the plaque area in mildly and severely thickened lesions. (**A**) In the mildly thickened lesion of Case 4, the CD163-stained area is larger than the CD80-stained area. (**B**) In the severely thickened lesions of Cases 1 and 3, the CD163-stained areas are larger than the CD80-stained areas.
