# Supplementary figures and images for: Preliminary Observations of Spatial Imbalance between Lymphangiogenesis and Angiogenesis within Carotid Atherosclerotic Plaques: A Pilot Histopathological Study
Source: Ann Vasc Dis. 2026 Apr 1;19(1):25-00157. doi: 10.3400/avd.oa.25-00157 (PMC13051298; doi:10.3400/avd.oa.25-00157)

Supplementary Fig. 1

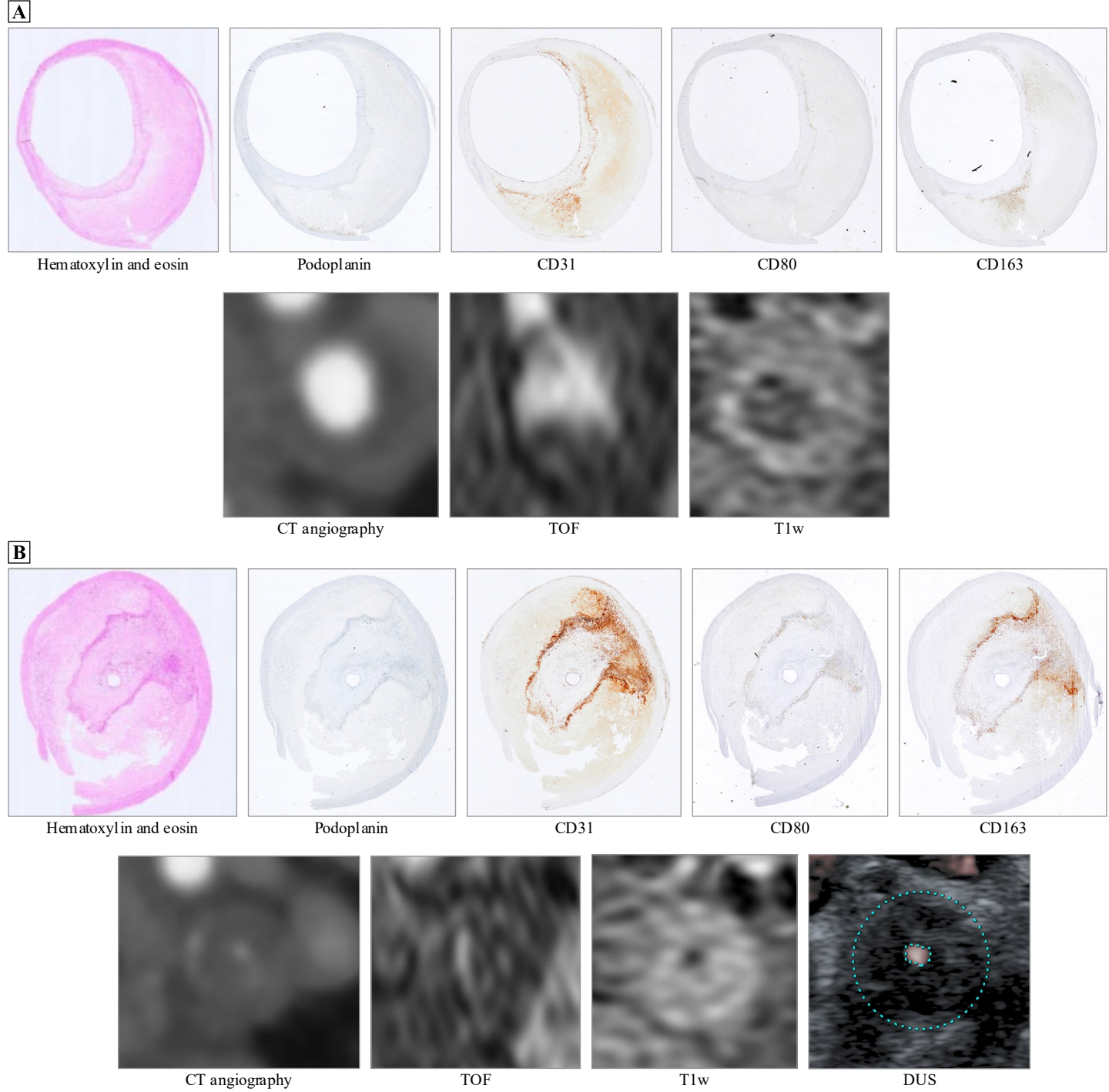

Supplementary Fig. 2

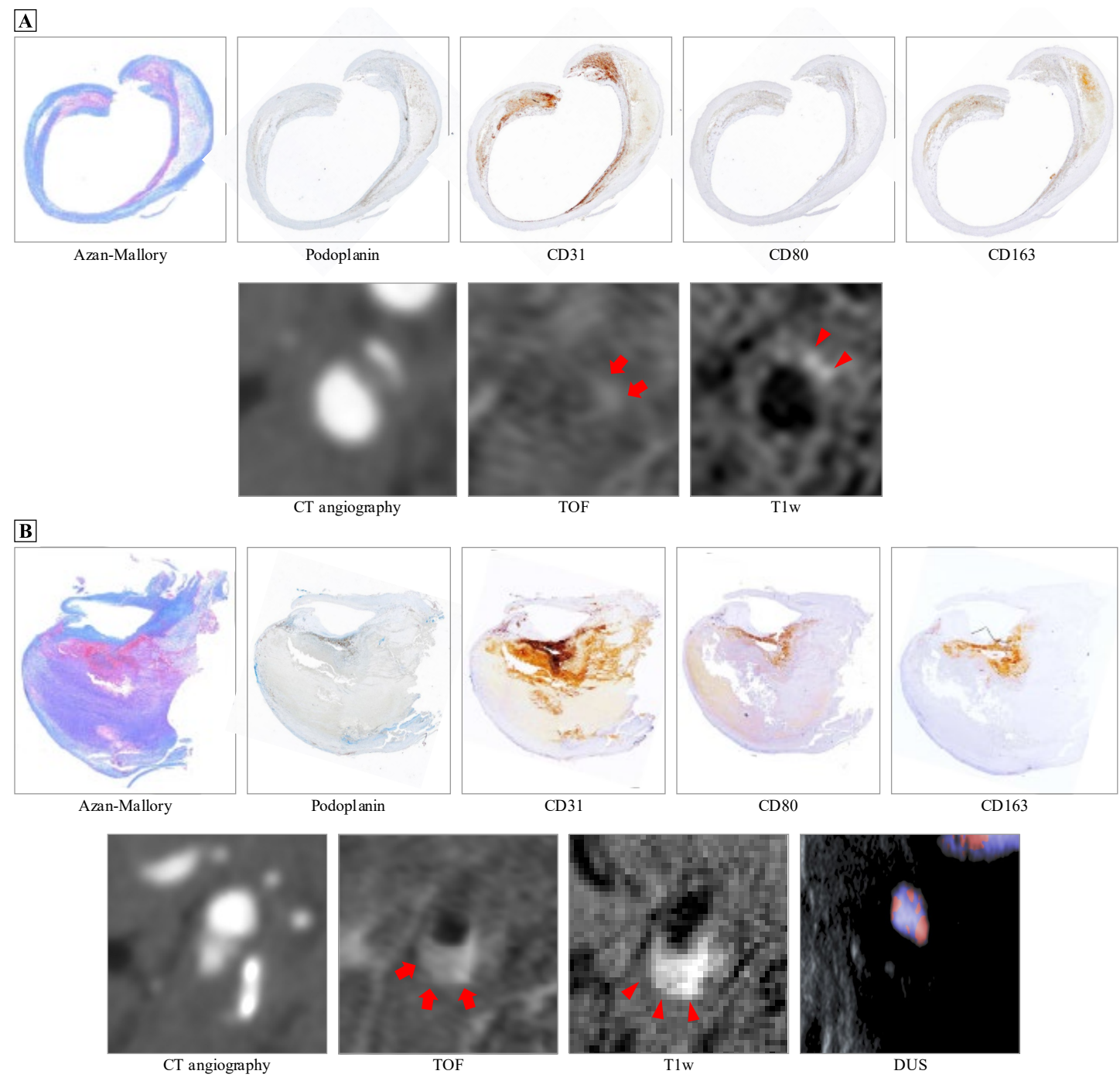

Supplementary Fig. 3

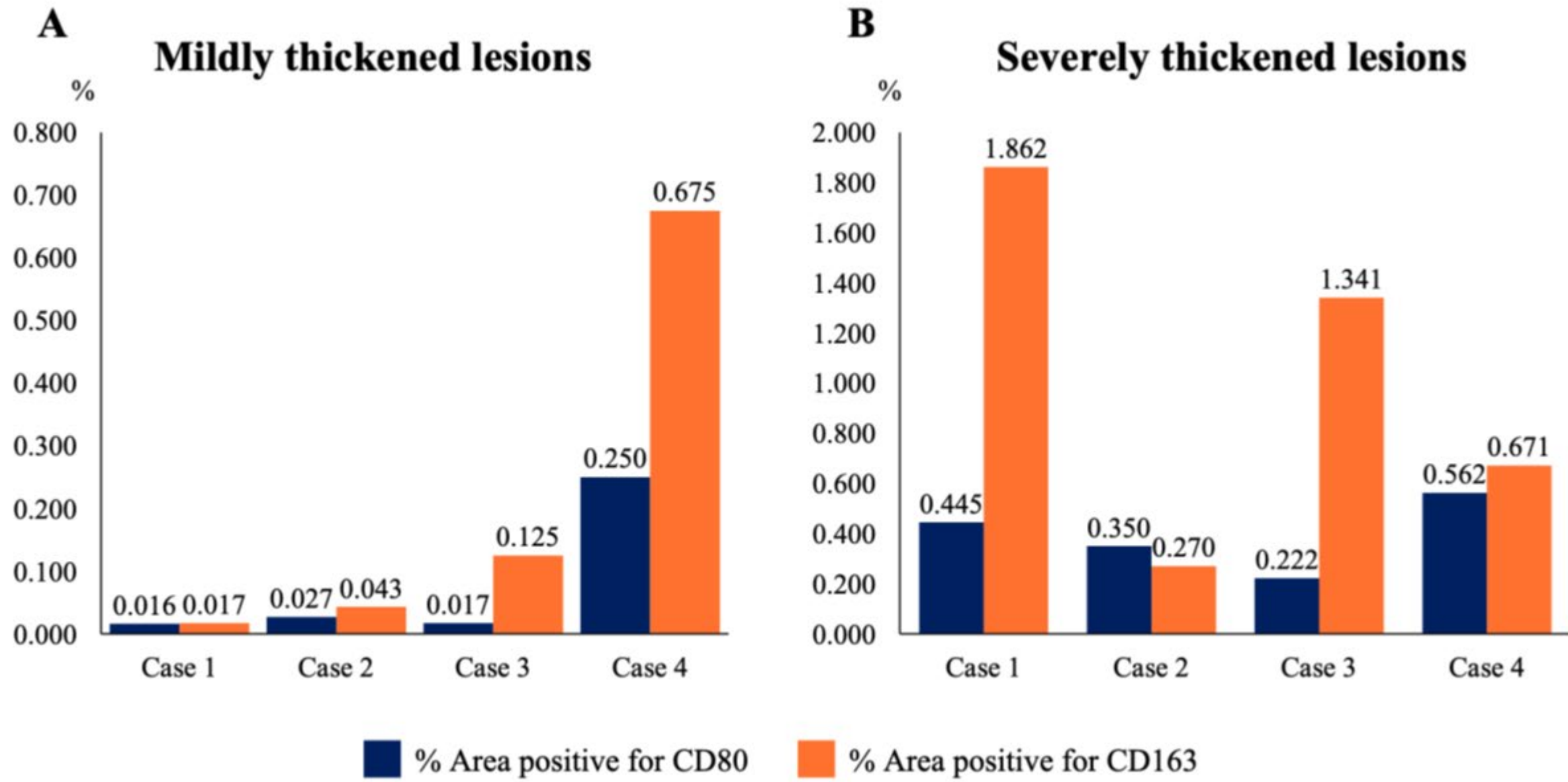

Supplement: Supplementary Fig. 1 — Histopathological and imaging findings of mildly and severely thickened lesions in Case 3. (A) The mildly thickened lesion contains a lipid-rich necrotic core, which appears slightly hyperintense on T1-weighted images. (B) In the severely thickened lesion, a lipid-rich necrotic core predominates, while intraplaque hemorrhage was minimal. The lesion appears slightly hyperintense on T1-weighted images and low intensity on time-of-flight images. CT: computed tomography; TOF: time-of-flight; T1w: T1-weighted; DUS: duplex ultrasonography [file avd-19-1-25-00157-s001.pdf]
